# Rare Functional Variants in Complement Genes and Anti-FH Autoantibodies-Associated aHUS

**DOI:** 10.3389/fimmu.2019.00853

**Published:** 2019-05-01

**Authors:** Elisabetta Valoti, Marta Alberti, Paraskevas Iatropoulos, Rossella Piras, Caterina Mele, Matteo Breno, Alessandra Cremaschi, Elena Bresin, Roberta Donadelli, Silvia Alizzi, Antonio Amoroso, Ariela Benigni, Giuseppe Remuzzi, Marina Noris

**Affiliations:** ^1^Clinical Research Center for Rare Diseases ‘Aldo e Cele Daccò’, Istituto di Ricerche Farmacologiche Mario Negri IRCCS, Bergamo, Italy; ^2^Azienda Ospedaliera–Universitaria, Città della Salute e della Scienza and Department of Medical Sciences, University of Turin, Turin, Italy; ^3^‘L. Sacco’ Department of Biomedical and Clinical Sciences, University of Milan, Milan, Italy

**Keywords:** autoantibodies, atypical hemolytic uremic syndrome, factor H, factor H related 1, complement, genetic variants, supercontrols

## Abstract

Atypical hemolytic uremic syndrome (aHUS) is a rare disease characterized by microangiopathic hemolytic anemia, thrombocytopenia and renal failure. It is caused by genetic or acquired defects of the complement alternative pathway. Factor H autoantibodies (anti-FHs) have been reported in 10% of aHUS patients and are associated with the deficiency of factor H-related 1 (FHR1). However, FHR1 deficiency is not enough to cause aHUS, since it is also present in about 5% of Caucasian healthy subjects. In this study we evaluated the prevalence of genetic variants in *CFH, CD46, CFI, CFB, C3*, and *THBD* in aHUS patients with anti-FHs, using healthy subjects with FHR1 deficiency, here defined “supercontrols,” as a reference group. “Supercontrols” are more informative than general population because they share at least one risk factor (FHR1 deficiency) with aHUS patients. We analyzed anti-FHs in 305 patients and 30 were positive. The large majority were children (median age: 7.7 [IQR, 6.6–9.9] years) and 83% lacked FHR1 (*n* = 25, cases) due to the homozygous *CFHR3-CFHR1* deletion (*n* = 20), or the compound heterozygous *CFHR3-CFHR1* and *CFHR1-CFHR4* deletions (*n* = 4), or the heterozygous *CFHR3-CFHR1* deletion combined with a frameshift mutation in *CFHR1* that generates a premature stop codon (*n* = 1). Of the 960 healthy adult subjects 48 had the FHR1 deficiency (“supercontrols”). Rare likely pathogenetic variants in *CFH, THBD*, and *C3* were found in 24% of cases (*n* = 6) compared to 2.1% of the “supercontrols” (*P*-value = 0.005). We also found that the *CFH* H3 and the *CD46*_*GGAAC*_ haplotypes are not associated with anti-FHs aHUS, whereas these haplotypes are enriched in aHUS patients without anti-FHs, which highlights the differences in the genetic basis of the two forms of the disease. Finally, we confirm that common infections are environmental factors that contribute to the development of anti-FHs aHUS in genetically predisposed individuals, which fits with the sharp peak of incidence during scholar-age. Further studies are needed to fully elucidate the complex genetic and environmental factors underlying anti-FHs aHUS and to establish whether the combination of anti-FHs with likely pathogenetic variants or other risk factors influences disease outcome and response to therapies.

## Introduction

Atypical hemolytic uremic syndrome (aHUS) is a rare disease characterized by microangiopathic hemolytic anemia, thrombocytopenia and renal failure (1, 2). It is mainly caused by genetic or acquired defects of the alternative pathway (AP) of the complement system (3). Heterozygous likely pathogenic variants (LPVs) in genes encoding complement regulators, such as factor H (FH), factor I and CD46 (3–7), or components of the AP C3 convertase (C3 and factor B) (3, 8–11) were found in about half of aHUS patients. Moreover, genetic abnormalities in thrombomodulin (*THBD*), an endothelial anticoagulant protein that also regulates complement, were reported in 3–5% of aHUS cases (12).

Incomplete penetrance was observed in carriers of genetic abnormalities, indicating that although LPVs predispose to the development of aHUS, additional genetic and/or environmental hits are necessary for the disease to manifest (13). Indeed, in a large multinational study, 25% of patients with LPVs in *CD46* or *CFI*, and 8–10% of those with LPVs in *CFH, C3*, and *CFB*, carried combined genetic abnormalities in other complement genes (13). Common variants and haplotypes in *CFH* and *CD46* have been associated with aHUS and were reported to increase the penetrance of the disease in subjects carrying LPVs (14–16).

Factor H autoantibodies (anti-FHs) have been described in about 10% of aHUS patients and are strongly associated with the deficiency of factor H-related 1 (FHR1) (17–19). In most cases, the lack of FHR1 is due to the polymorphic homozygous deletion of the factor H related 3 and 1 genes (*CFHR3-CFHR1*) (20). In about 15% of patients the FHR1 deficiency is caused by compound heterozygous deletion of *CFHR3-CFHR1* and *CFHR1-CFHR4*, while homozygous *CFHR1-CFHR4* deletion is rare (21–23).

The pathogenic mechanism that links the FHR1 deficiency with the risk of the development of anti-FHs has not been clarified yet. Anti-FHs mainly target the C-terminal region of FH, and also cross react with FHR1 short consensus repeats (SCRs) 4–5, which have very high amino acid sequence identity with SCRs 19–20 of FH (24–27). It has recently been hypothesized that binding of certain proteins expressed by pathogens to the SCRs 19–20 of FH induces the exposure of a neoepitope in FH that is conformationally similar to that in SCRs 4–5 of FHR1, resulting in an autoimmune response in subjects with FHR1 deficiency (26).

Despite the above evidence, FHR1 deficiency is not enough to cause anti-FH associated aHUS, which is an ultra-rare disease with an estimated prevalence of 1:1,000,000 (1, 28). Indeed, the *CFHR3-CFHR1* deletion is a rather common polymorphism, and is present in homozygosity in 3–10% of healthy European populations and in 7–30% of African populations, whereas it is very rare in Asia and South America (29). The combined *CFHR3-CFHR1* and *CFHR1-CFHR4* deletion is present in 0.9% of healthy European controls (22).

Autoimmune diseases are typically complex disorders in which multiple susceptibility genetic variants and environmental factors are involved (30, 31). The contribution of each genetic variant is usually small and only the presence of multiple variants favors the development of the disease (31). Notably, each single risk variant may be found in healthy subjects, although at a lower frequency than in patients, but it is the combination of multiple risk variants that clusters with the disease (30).

Previous studies reported that about 14.7% of aHUS patients with anti-FHs also carry LPVs in complement genes (18, 19, 21–23, 32); however the prevalence of LPVs in patients with anti-FHs should be compared to that in the general population or, even better, in a control group with FHR1 deficiency. This would support the hypothesis that LPVs have a role in anti-FHs aHUS.

In this study we investigated the genetic determinants of anti-FH-associated aHUS by comparing patients with anti-FHs and FHR1 deficiency (defined here as “cases”) and adult healthy subjects carrying the homozygous *CFHR1* deletion (defined here as “supercontrols”) as a homogeneous, special control group, to optimize the power to detect differences. We hypothesized that the “supercontrols” were depleted for risk variants or even enriched in protective variants, since they evaded the development of aHUS despite carrying the known risk factor represented by the FHR1 deficiency.

Specifically, we compared the prevalence of rare LPVs in complement genes between cases and “supercontrols.” We also investigated the association with anti-FHs aHUS of common variants in complement genes, which in previous studies were reported to increase the risk of aHUS or of other immune-mediated glomerulopathies (33–38).

We found that rare LPVs but not common complement gene variants are enriched to a significant extent in cases compared to “supercontrols,” suggesting they are involved in the development of anti-FHs aHUS.

## Methods

### Patients and Controls

In all patients, atypical HUS was diagnosed based on microangiopathic hemolytic anemia and thrombocytopenia defined as hematocrit < 30%, hemoglobin level < 10 g/dl, serum lactate dehydrogenase level above 500 U/l, undetectable haptoglobin level, fragmented erythrocytes in peripheral blood smear, and platelets below 150 × 10^3^/μl, associated with acute renal failure (serum creatinine >1.3 mg/dl for adults, >0.5 mg/dl for children under 5 years of age and >0.8 mg/dl for children aged 5–10 years old; and/or urinary protein/creatinine ratio >200 mg/g; or an increase of serum creatinine or urinary protein/creatinine ratio >15% compared to baseline levels). All aHUS patients (*n* = 305) were recruited through the International Registry of HUS/TTP (Ranica, Bergamo, Italy).

Healthy adult subjects (*n* = 960) were from internal donors and from the Piedmont Regional Registry of the Italian Bone Marrow Donors Registry (Azienda Ospedaliera–Universitaria, Città della Salute e della Scienza, Turin, Italy). Patient and healthy subject data were handled with respect for confidentiality and anonymity. All subjects provided informed written consent in accordance with the Declaration of Helsinki. The study was approved by the Ethics Committee of Azienda Sanitaria Locale, Bergamo, Italy.

### Anti-FH IgG ELISA

The presence of anti-FH IgGs was evaluated through an Enzyme-Linked Immunosorbent Assay (ELISA). Microtiter plates were coated with 100 μl of a solution of 1 μg/ml of purified human FH (0.1 μg, Calbiochem). After overnight incubation at 4°C, the plate was washed with Phosphate-Buffered Saline (PBS), 0.1% Tween20, and 0.2 M NaCl and blocked with PBS, 0.1% Tween 20, and 0.3% milk powder. After washes, 50 μl of plasma/serum samples at 1:100 dilution were added to duplicated wells, and incubated for 40 min. A parallel plate was set up in the absence of FH coating, in which only blocking solution was added to evaluate the presence of non-FH specific background, according to published recommendations (39). After washes, goat anti-human IgG antibody conjugated with horseradish peroxidase (HRP, 1:250, Sigma-Aldrich) was added and incubated for 1 h. Enzymatic activity was revealed using the 3,3′,5,5′-tetramethylbenzidine (TMB) substrate. A positive control kindly gifted by Dr. Marie-Agnes Dragon-Durey, with 2,000 AU/ml titer was used at 1:100 dilution. The positive threshold was set up at the mean titer +2 standard deviations (SD) found in plasma samples of 98 healthy controls with 1 or 2 copies of *CFHR1* (56 AU/ml). The sample concentrations expressed in arbitrary units/ml (AU/ml) were extrapolated from a sigmoidal curve and the background derived from wells without FH coating was subtracted. The ELISA was repeated in positive samples, adding 15 μg of FH into each sample to verify antibody specificity. Samples showing at least a 50% decrease of the ELISA absorbance in the assay performed in the presence of exogenous FH were considered as true positive.

This ELISA protocol was also used to evaluate the binding of autoantibodies to SCRs 1–5 and 15–20 FH fragments obtained by baculovirus transfection of *Spodoptera frugiperda* cells as reported (40). The plate was coated with molar equivalents of FH SCRs 1–5 and 15–20 and the results were expressed as absorbance at 450 nm. In a selected experiment, additional wells were coated with full length FH (positive control) or bovine serum albumin (BSA, negative control).

### HMEC-1

Serum-induced C5b-9 deposition on a human microvascular endothelial cell line (HMEC-1) was analyzed as previously described, with minor modifications (41). HMEC-1 were plated on glass coverslips and used when confluent. Cells were activated with 10 μM ADP for 10 min and incubated for 4 h with serum from patients (ID: 18 and 20) or healthy controls diluted 1:2 with test medium (Hank's Balanced Salt Solution—HBSS +0.5% BSA). HMEC-1 were fixed and stained with rabbit anti-human C5b-9 antibody, followed by FITC-conjugated antibody. Fluorescent staining on cell surface was acquired in 15 fields through confocal microscopy and the staining area was evaluated using Image J software. Results were expressed as the percentage of staining in relation to a pooled control sera (*n* = 10 subjects) run in parallel. The sera from additional 35 controls were analyzed separately and the percentages of C5b-9 deposits vs. control serum pool were calculated to establish the normal range (mean ± 2SD of % C5b-9 deposits of the control sera vs. control serum pool: 60–149%).

### FH ELISA

FH levels were evaluated by ELISA. Microtiter plates were coated with 0.15 μg of purified sheep polyclonal anti-human factor H antibody (Abcam). After three washes with PBS, 0.05% Tween20, the plate was blocked with 200 μl of PBS, 1% BSA. After four washes with PBS, 0.05% Tween20, 100 μl of plasma samples at 1:10,000 dilution were added to duplicated wells, and incubated for 2 h. After the washes, 100 μl of mouse monoclonal anti-human factor H (OX-23, LifeSpan BioSciences), which recognizes the SCRs 1–4 of the FH N-terminal domain, were added at a 1:10,000 dilution in blocking solution. Notably, this anti-human factor H antibody also detected the FH-like 1 (FHL1) which shares the N-terminal domain with FH. The plate was washed and 100 μl of goat anti-mouse antibody conjugated with HRP (1:2,000, Thermo Fisher) were added and incubated for 1 h. Enzymatic activity was revealed using the TMB substrate.

### SDS-PAGE and Western Blotting

FHR1 was studied by Western blotting in the serum of all patients with anti-FHs with at least one copy of *CFHR1*. Sera were diluted 1:40 in loading buffer (4X Laemmli Sample Buffer, Bio-Rad) in non-reducing conditions and analyzed using sodium dodecyl sulfate-polyacrylamide gel electrophoresis (SDS-PAGE, Mini-Protean TGX Precast Gels, Bio-Rad). Proteins were transferred to polyvinylidene difluoride membrane (PVDF, Trans-Blot Turbo Midi PVDF Transfer; Bio-Rad), and blocked with 4% skim milk and 1% BSA. Mouse anti-human FHR1 IgGs (1:1,000, kindly given by Prof. Peter Zipfel) (42), which recognizes the N-terminal domains of FHR1, were added for 1 h. The binding of the antibody was detected by 1 h incubation with HRP goat anti-mouse IgG (1:5,000), followed by enhanced chemiluminescence (ECL) substrate (Amersham).

### Measurement of *CFH* and *CFHRs* Copy Number

Multiplex-ligation dependent probe amplification (SALSA MLPA P236-A3 ARMD, MCR-Holland, Netherlands) was used to evaluate the presence of copy number variations (CNVs) in *CFH, CFHR3, CFHR1, CFHR2, CFHR5* genes in the 30 aHUS patients with anti-FHs. The *CFHR4* CNV was evaluated in aHUS patients with anti-FHs by a multiplex PCR, as reported by Moore et al. (22). To evaluate the presence of the homozygous deletion of *CFHR1* in all 305 aHUS patients, and in the 960 healthy adult controls a multiplex PCR was set up that amplifies a 133 bp fragment in intron 3 of *CFHR1* and a 83 bp fragment in the promoter of *RNAseP*.

Primers: *CFHR1*-For 5′-ATCACTACACATGGACCTGAAA-3′; *CFHR1*-Rev 5′-GATGTGGAAAAATAAAAGAAAATAAGTC-3′; *RNAseP*-For 5′-TAGATACCGTGTGCGTGCAT-3′; *RNaseP*-Rev 5′-GGGGTTCCAATTCCCAACTA-3′.

### Genetic Screening

Through next-generation sequencing we performed genetic screening of all exons and flanking regions of *CFH, CD46, CFI, CFB, C3*, and *THBD* by highly multiplex PCR using the Ion AmpliSeq™ Library Kit on an Ion PGM Sequencer (Life Technologies) (34).

LPVs were defined as genetic variants with Minor Allelic Frequency (MAF) in the ExAC database ≤ 0.001 and a Combined Annotation Dependent Depletion (CADD) pathogenic score ≥20 (43). Considering the complexity of aHUS and the very low penetrance we also considered as likely pathogenic, variants with a MAF ≤ 0.01 for which there was evidence of functional effects in literature. In addition, we included as likely pathogenic, genetic variants with MAF ≤ 0.01 for which an association with aHUS was reported (*P*-value < 0.001, vs. “ExAC all” in the database of complement gene variants, www.complement-DB.org).

The *CFH* promoter and the coding and flanking regions of *CFHR1* were analyzed by direct sequencing.

### Statistical Analyses

All statistical tests were executed using MedCalc software. The Chi-square test or the Fisher's exact test were used to compare the allele frequencies between cases and “supercontrols,” as appropriate. The expectation maximization algorithm by Haploview software was used to estimate each *CFH* haplotype frequency. Findings were considered statistically significant at *P*-values < 0.05 after Bonferroni correction. Odds ratio (OR) was reported with the 95% confidence interval. ANOVA or Kruskal-Wallis tests were used to compare the mean or the median of variables, as appropriate.

## Results

### Factor H Autoantibodies

**Figure 1:**
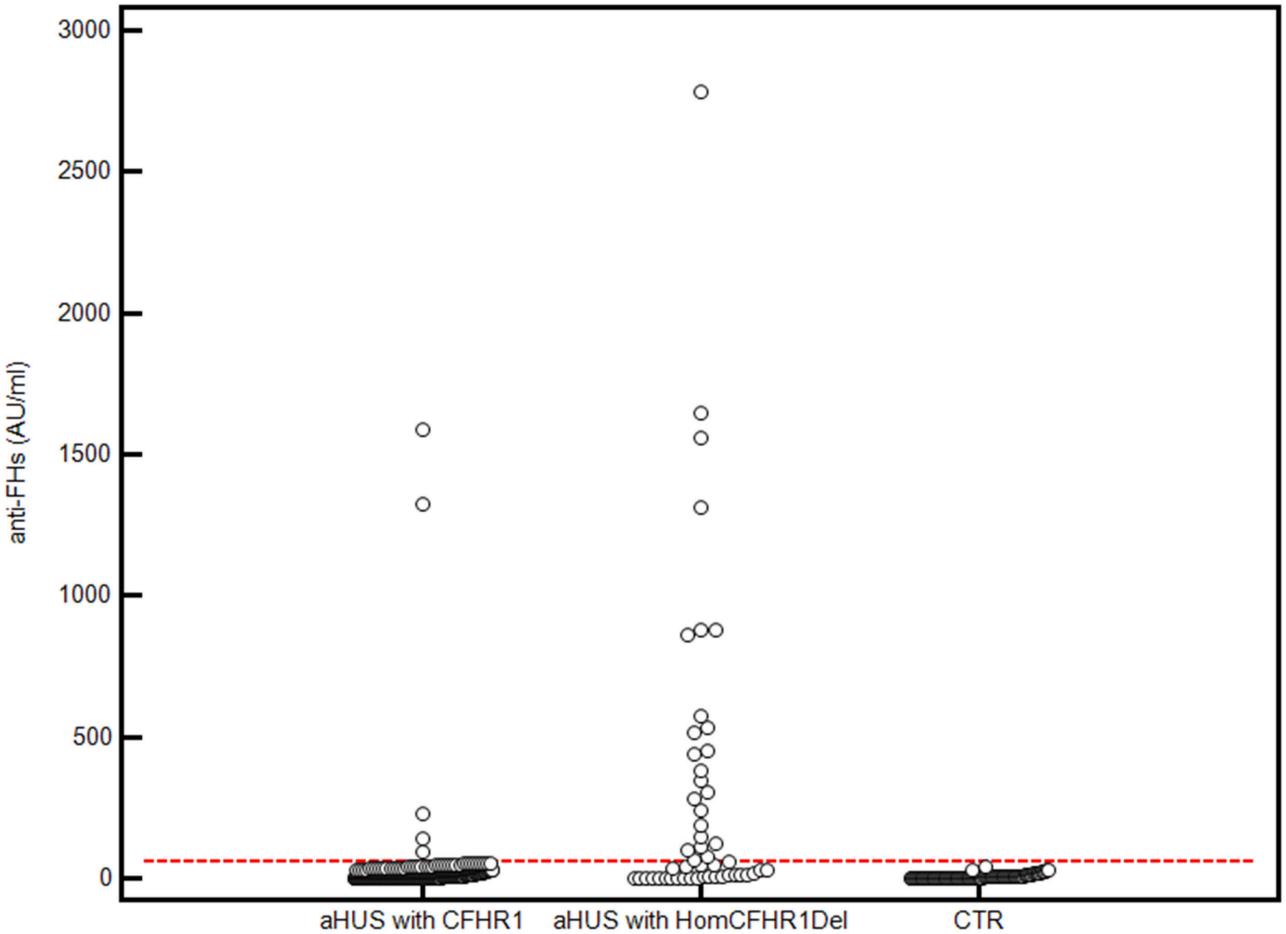
Anti-FHs in aHUS patients and healthy controls. 305 aHUS patients were analyzed for anti-FHs through ELISA: 254 were carriers of 1 or 2 copies of *CFHR1* (aHUS with *CFHR1*), and 51 had 0 copies of *CFHR1* (aHUS with Hom*CFHR1*Del). Patient 23, carrying the heterozygous *CFHR1*Del and a frameshift variant in *CFHR1* exon 2 (c.104delAfsX), which resulted in a complete deficiency of FHR1 has been also included in the group of patients defined as “aHUS with Hom*CFHR1*Del.” Ninety-eight healthy controls with 1 or 2 copies of *CFHR1* were also analyzed for anti-FHs (CTR). The positive threshold was set at mean +2SD of values recorded in the 98 controls (56 AU/mL) and is shown with the horizontal red dashed line.

The presence of anti-FHs was assessed in 305 consecutive aHUS patients of the International Registry of HUS for whom serum or plasma was available. Thirty patients (9.8%) were positive for anti-FHs (anti-FHs titer threshold >56 AU/ml); in all of them the specificity of the result was confirmed in a replicated assay, in which an excess of FH was added to the serum/plasma sample and competed for the anti-FHs with FH coated on the well (Figure 1). The mean ± SD of anti-FH titers was 1,164.7 ± 1,189.5 AU/ml in samples collected during the acute phase of the disease (*n* = 4) and 521.8 ± 504.3 AU/ml in samples collected during remission (*n* = 26; ANOVA, *P*-value = 0.062).

### FHR1 Deficiency

In all 30 patients with anti-FHs aHUS, copy numbers were evaluated by MLPA for *CFH, CFHR3, CFHR1, CFHR2*, and *CFHR5* and by a multiplex PCR for *CFHR4*. The homozygous *CFHR3-CFHR1* deletion was observed in 20 patients (66.7%) and the heterozygous *CFHR3-CFHR1* deletion combined with the heterozygous *CFHR1-CFHR4* deletion was present in 4 patients (13.3%, Table 1). Overall, 24 patients had zero copies of *CFHR1* (80%, Table 1). Four patients showed the heterozygous deletion of *CFHR3* and *CFHR1* (13.3%) and in two patients no CNV in *CFHR* genes were identified (6.7%, Table 1).

**Table 1:** Copy numbers of *CFHR3, CFHR1*, and *CFHR4* and LPVs in complement genes observed in aHUS patients with anti-FHs.

**Patient ID**	**Number of copies**			**LPVs**	**MAF (ExAC)**	**CADD**	**Functional effect**	**Association with aHUS (complement-db.org)**
	**CFHR3**	**CFHR1**	**CFHR4**					
1	0	0	2	nd				
2	0	0	2	*CFH* c.2850G>T, p.Gln950His	0.004	21.4	Yes	No
3	0	0	2	nd				
4	1	0	1	nd				
5	0	0	2	nd				
6	0	0	2	*C3* c.1909G>C, p.Gly637Arg	0.0002	23.5	No	No
7	0	0	2	*THBD* c.1693 G>T, p.Asp486Tyr	0.006	6.12	Yes	No
8	0	0	2	nd				
9	0	0	2	nd				
10	0	0	2	nd				
11	0	0	2	nd				
12	0	0	2	nd				
13	1	1	2	*CD46* c.762delT, p.Leu254fsX43	na	17.57	Yes	No
14	0	0	2	nd				
15	0	0	2	nd				
16	1	0	1	nd				
17	0	0	2	nd				
18	0	0	2	nd				
19	1	1	2	*THBD* c.1502C>T, p.Pro501Leu	0.002	24.7	Yes	Yes
20	0	0	2	nd				
21	2	2	2	*C3* c.1774C>T, p.Arg592Trp	8 × 10^−6^	34	Yes	Yes
22	0	0	2	nd				
23	1	1^*^	2	nd				
24	0	0	2	*CFH* c.2758T>C, p.Trp920Arg	na	27	No	Yes
25	0	0	2	nd				
26	1	1	2	nd				
27	0	0	2	*CFH* c.2776T>C, p.Cys926Arg	na	28.4	No	No
28	1	0	1	nd				
29	1	0	1	*THBD* c. 241G>A, p.Val81Ile	9 × 10^−6^	8.28	Yes	Yes
30	2	2	2	nd				

The Minor Allelic Frequency (MAF) of LPVs from the ExAC database, the Combined Annotation Dependent Depletion (CADD) score, the availability of published data on functional effects, and the association with aHUS in the database of complement gene variants (complement-db.org) are also shown. Nd, not detected; na, not available.

* *The CFHR1 c.104delAfsX, p.D35fsX36 was observed in this patient*.

**Figure 2:**
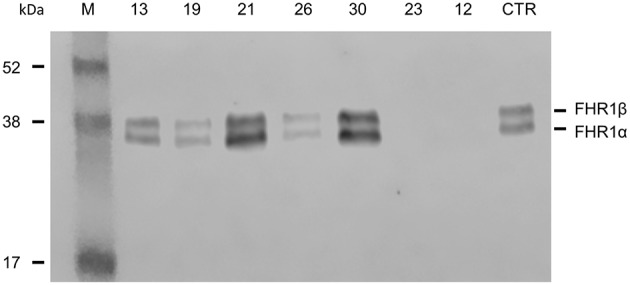
Detection of FHR1 by Western blotting in aHUS patients with anti-FHs and at least one copy of *CFHR1*. ID numbers 13, 19 and 26: patients with 1 copy of *CFHR1*; ID numbers 21 and 30: patients with 2 copies of *CFHR1*; ID number 23: patient with 1 copy of *CFHR1* and a frameshift mutation in exon 2 (c.104delAfsX); ID number 12: patient with 0 copies of *CFHR1*; CTR: an healthy control with two copies of *CFHR1*.

Direct sequencing of *CFHR1* was performed in the 6 patients with anti-FHs and at least one copy of *CFHR1*. In patient 23, who carried the heterozygous *CFHR1* deletion, a frameshift variant in *CFHR1* exon 2 (c.104delAfsX) that resulted in a premature stop codon (p.D35fsX36) was observed. In all six patients, the presence of FHR1 in serum was verified by Western blot (Figure 2). The normal pattern of two FHR1 bands was observed in patients 13, 19, and 26 (all with one copy of *CFHR1*) and patients 21 and 30 (both with two copies of *CFHR1*). At variance, patient 23, who had one copy of *CFHR1* plus the c.104delAfsX frameshift mutation did not have any FHR1 band, confirming that the mutation resulted in a non-secreted truncated protein, as expected. Thus, in our cohort, 25/30 (83.3%) patients with anti-FHs showed a complete deficiency of FHR1 and are defined as cases here.

We also evaluated the prevalence of the acidic and basic FHR1 isoforms in patients with anti-FHs and with at least one copy of *CFHR1*. In the basic FHR1 isoform that has been associated with the risk of aHUS (21), the amino acids 157His, 159Leu, and 175Glu are replaced by 157Tyr, 159Val, and 175Gln, which make the SCR3 of FHR1 identical to the SCR18 of FH. In patients with the heterozygous *CFHR3-CFHR1* deletion, one exhibited the basic isoform and two the acidic isoform. Both patients with two copies of *CFHR3* and *CFHR1* were compound heterozygous for the two FHR1 isoforms. Thus, we did not find an enrichment of a specific FHR1 isoform in our aHUS patients with anti-FHs.

A multiplex PCR was used to identify subjects with the *CFHR1* homozygous deletion in all 305 unrelated aHUS patients analyzed for anti-FHs and in a large cohort of healthy subjects (*n* = 960). Overall, 49 aHUS patients (16%) and 48 healthy subjects (5%) were found to be homozygous for the *CFHR1* deletion (OR [95%CI] = 3.6 [2.4–5.5], *P*-value = 5.5 × 10^−10^, Table 2). The association of the homozygous *CFHR1* deletion with aHUS strongly increased in the subgroup of patients with anti-FHs vs. healthy controls (OR [95%CI] = 76 [29.7–194.6]*, P*-value = 3 × 10^−52^, Table 2).

**Table 2:** Prevalence of homozygous *CFHR1* deletion in aHUS patients (*n* = 305) and healthy controls (*n* = 960).

	**Hom*CFHR1*Δ**	**No Hom*CFHR1*Δ**	**OR [95%CI]**	***P*-value**
aHUS (*n* = 305)	49 (16%)	256 (84%)	3.6 [2.4–5.5]	5.5 × 10^−10^
Controls (*n* = 960)	48 (5%)	912 (95%)		
aHUS anti-FH positive (*n* = 30)	24 (80%)	6 (20%)	76 [29.7–194.6]	3 × 10^−52^
Controls (*n* = 960)	48 (5%)	912 (95%)		

### Binding of Anti-FHs to FH Fragments and Effect of Patient Sera on C5b-9 Deposition on HMEC-1

**Figure 3:**
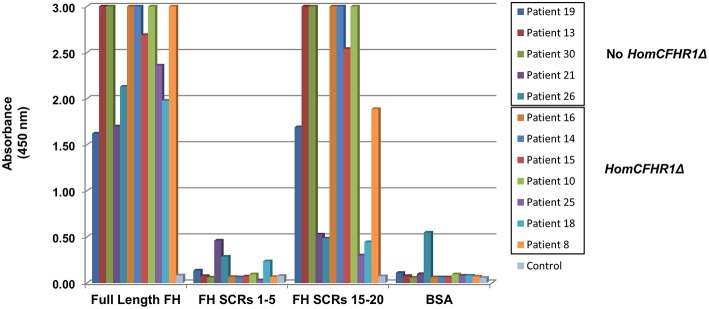
Binding site localization of anti-FHs detected in aHUS patients. Anti-FH binding to FH N-terminal fragment (SCRs 1–5) and FH C-terminal fragment (SCRs 15–20) evaluated in 12 aHUS patients. Seven patients were carriers of the homozygous *CFHR1* deletion (Hom*CFHR1*Δ) while 5 patients presented at least one copy of *CFHR1* (No Hom*CFHR1*Δ). BSA coating was used as negative control and full length FH coating as positive control. Absorbance of serum from a healthy subject was used as an additional control. The absorbance is shown on the ordinate axis. Data are representative of three experiments.

To discern which FH epitopes were recognized by anti-FHs, anti-FH ELISA was repeated using wells coated with N-terminal FH (SCRs 1–5) and C-terminal FH (SCRs 15–20) fragments (kindly given by Prof. Peter Zipfel). The results of samples from five patients with 1 or 2 copies of *CFHR1* (patients 19, 13, 30, 21, and 26) and from seven patients carrying the homozygous *CFHR1* deletion (patients 16, 14, 15, 10, 25, 18, and 8) are shown in Figure 3. Antibodies from patients 13, 19, and 30 (with 1, 1 and 2 copies of *CFHR1*, respectively) and from patients 8, 10, 14, 15, and 16 (with 0 copies of *CFHR1*) strongly bound to C-terminus but not to N-terminus of FH. In patient 25 with the homozygous deletion of *CFHR1*, we observed a less intense but selective binding to FH C-terminal fragment. Antibodies from patients 18 (with the homozygous deletion of *CFHR1*) and 21 (with two copies of *CFHR1*) bound weakly to both FH SCRs 1–5 and FH SCRs 15–20 and finally, antibodies from patient 26 (with one copy of *CFHR1*) did not specifically bind any FH fragments. It is possible that autoantibodies from patient 26 recognize either central FH epitopes or epitopes exposed only in the native, full length FH, as previously reported for other aHUS patients (22).

**Figure 4:**
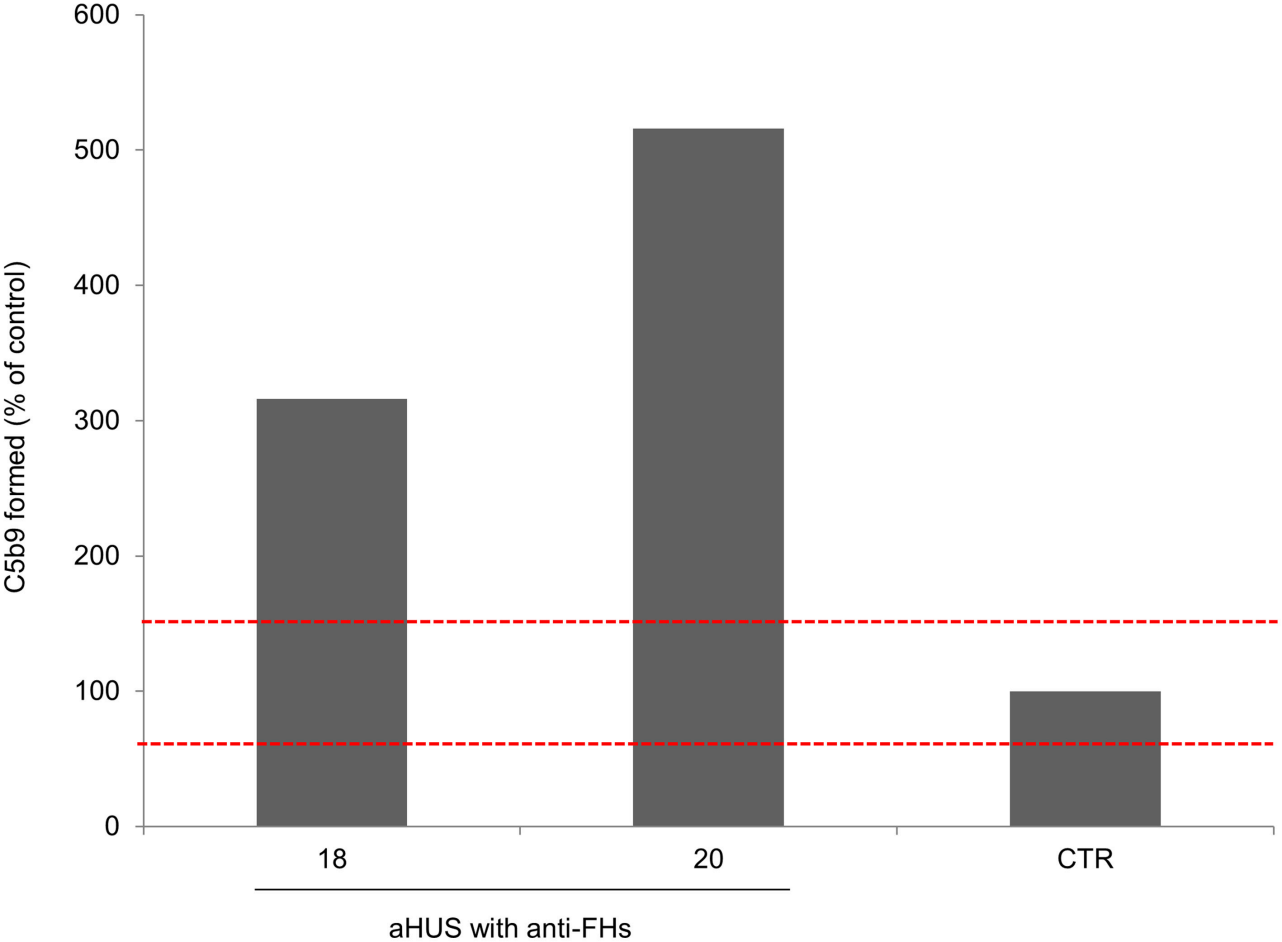
Complement activation on endothelial cells. Endothelial surface area covered by C5b-9 staining after incubation of ADP-activated HMEC-1 with serum from aHUS patients with anti-FHs studied at remission (patients 18 and 20). For each sample, values were expressed as the percentage of C5b-9 deposits induced by a pool of sera from 10 healthy controls run in parallel (reference 100%). The red dashed lines indicated the normal range (60–149%) determined by testing single sera from 35 different healthy controls.

An *ex-vivo* test to evaluate complement activation at the endothelial cell level showed that serum from two patients (ID: 18 and 20, without LPVs and with homozygous *CFHR1* deletion) taken after aHUS remission induced higher than normal (>149%) C5b-9 deposits on cultured ADP-activated human microvascular endothelial cells (Figure 4).

### Complement Likely Pathogenic Variants (LPVs)

*CFH, CD46, CFI, CFB, C3*, and *THBD* were sequenced in all patients with anti-FHs and 9 were found to be carriers of LPVs as defined in methods (30%, Table 1).

**Figure 5:**
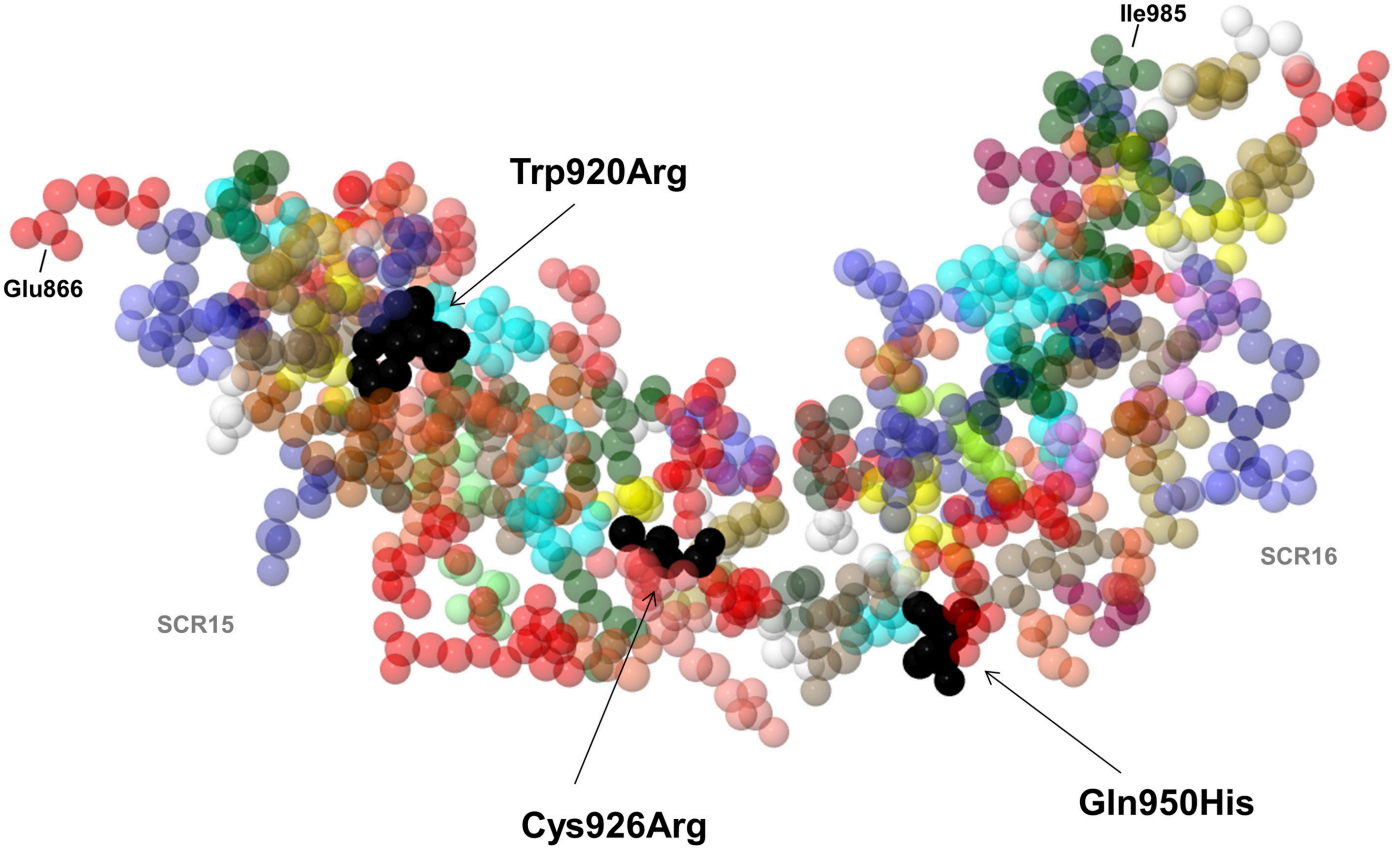
3D structure by nuclear magnetic resonance of SCR15 and SCR16 of FH (PDB code: 1HFH). Black arrows indicate the FH amino acid residues Trp920, Cys926 (SCR15), and Gln950 (SCR16) (colored in black) which are substituted in three aHUS patients with anti-FHs due to the Trp920Arg, Cys926Arg, and Gln950His heterozygous mutations. Each amino acid is represented with a specific color. The first amino acid of SCR15 (Glu866) and the last amino acid of SCR16 (Ile985) are also indicated. Cysteine residues are shown in yellow color. Created by Jmol, an open-source Java viewer for chemical structures in 3D (http://www.jmol.org).

Of the 25 patients with anti-FHs and the FHR1 deficiency (cases), 6 had LPVs (24%, Table 1). Three cases carried LPVs in *CFH* (12%). Two Italian cases showed the heterozygous *CFH* LPV c.2850G>T, p.Gln950His in SCR16 (rs149474608, MAF (ExAC) = 0.004, CADD = 21.4) and the c.2758T>C, p.Trp920Arg (CADD = 27) in SCR15, respectively. These two patients had been previously reported by our group (3). Moreover, the p.Gln950His variant was previously described in another aHUS patient with anti-FHs (22), in other aHUS patients without anti-FHs (44–46), and in a patient who had developed TMA after renal transplantation (47). The third case was a Jewish patient who showed the heterozygous *CFH* c.2776C>T, p.Cys926Arg (CADD = 28.4) in SCR15. This variant is not present in public databases and has not been described in aHUS patients before. Notably, nuclear magnetic resonance studies (48) showed that the 3 LPVs involve amino acids that located close to each other in the three-dimensional structure of SCRs 15 and 16 of FH (Figure 5).

In an Italian case we found the C3 heterozygous c.1909G>C, p.Gly637Arg (rs149850773, MAF (ExAC) = 0.0002, CADD = 23.5), which is located in the Linker domain of the C3 molecule and has not been reported in patients with aHUS before.

We identified two *THBD* heterozygous LPVs: the c.1693G>T, p.Asp486Tyr (rs41348347, MAF (ExAC) = 0.006, CADD = 6.12) was identified in an Italian case and the c.241G>A, p.Val81Ile (rs772288987, MAF (ExAC) = 9 × 10^−6^, CADD = 8.28) was found in a case from Yemen. Both *THBD* variants have already been reported in patients with aHUS (3, 49).

Of the 48 adult healthy “supercontrols” with the homozygous deletion of *CFHR1*, only one carried a LPV, namely the heterozygous *CFI* c.949 G>A, p.Arg317Trp [rs121964917, MAF (ExAC) = 9.9 × 10^−5^, CADD = 16.27]. The FI 317Trp variant has previously been reported to have 30% C3b and C4b cofactor activity compared to wild type FI, but conflicting results have been found by other authors (50, 51).

Overall, in cases the prevalence of LPVs was significantly higher than in “supercontrols” (24 vs. 2.1%, Fisher's exact test, *P*-value = 0.005).

### Common Complement Susceptibility Variants

We then investigated whether common genetic variants in *CFH* contribute to determining susceptibility to anti-FHs mediated aHUS by comparing the prevalence of *CFH* c.1-332C>T (rs3753394), c.184G>A p.Val62Ile (rs800292), c.1204T>C p.Tyr402His (rs1061170), c.2016A>G Gln572Gln (rs3753396), c.2237-543G>A (rs1410996), c.2808G>T Glu936Asp (rs1065489) in the 25 cases and in the 48 “supercontrols.” As shown in Table 3, allele frequencies did not differ between cases and “supercontrols.”

**Table 3:** Prevalence of common variants in *CFH, CD46, CFB, C3*, and *THBD* in cases (*n* = 25) and “supercontrols” (*n* = 48).

	**Genotype**			**Sum**	**Allele frequencies**		***P*-value**
	**Hom WT**	**Het**	**Hom Var**		**WT**	**Var**	
***CFH*** **c.1-332C>T (rs3753394)**							
Cases	7	13	5	25	0.54	0.46	ns
Supercontrols	19	26	3	48	0.67	0.33	
***CFH*** **c.184G>A Val62Ile (rs800292)**							
Cases	20	5	0	25	0.90	0.10	ns
Supercontrols	42	6	0	48	0.94	0.06	
***CFH*** **c.1204T>C Tyr402His (rs1061170)**							
Cases	22	2	0	24	0.96	0.04	ns
Supercontrols	47	1	0	48	0.99	0.01	
***CFH*** **c.2016A>G Gln572Gln (rs3753396)**							
Cases	22	3	0	25	0.94	0.06	ns
Supercontrols	46	2	0	48	0.98	0.02	
***CFH*** **c.2237-543G>A (rs1410996)**							
Cases	0	4	21	25	0.08	0.92	ns
Supercontrols	0	2	46	48	0.02	0.98	
***CFH*** **c.2808G>T Glu936Asp (rs1065489)**							
Cases	22	3	0	25	0.94	0.06	ns
Supercontrols	47	1	0	48	0.99	0.01	
***CD46*** c.*783T>C (rs7144)							
Cases	10	11	4	25	0.62	0.38	ns
Supercontrols	19	24	5	48	0.65	0.35	
***CFB*** **Arg32Trp/Gln (rs12614, rs641153)**							
Cases	15	9	1	25	0.78	0.22	ns
Supercontrols	33	15	0	48	0.84	0.16	
***C3*** **c.941C>T Pro314Leu (rs1047286)**							
Cases	17	8	0	25	0.84	0.16	ns
Supercontrols	33	13	2	48	0.82	0.18	
***C3*** **c.304C>G Arg102Gly (rs2230199)**							
Cases	14	11	0	25	0.78	0.22	ns
Supercontrols	33	13	2	48	0.82	0.18	
***THBD*** **c.1418C>T Ala473Val (rs1042579)**							
Cases	16	9	0	25	0.82	0.18	ns
Supercontrols	37	11	0	48	0.89	0.11	

*Hom, homozygous; WT, wild type; Het, heterozygous; Var, variant; ns, not significant. For the CFB Arg32Trp/Gln triallelic SNP “var” includes both Trp and Gln variants. Cases: aHUS patients with anti-FHs and FHR1 deficiency; “supercontrols”: adult healthy subjects with homozygous CFHR1 deletion*.

*CFH* haplotypes were also studied in cases and “supercontrols.” The minimal informative SNPs within the *CFH* gene considered for this analysis were rs3753394 (promoter), rs800292 (exon 2), rs1061170 (exon 9), rs3753396 (exon 14), rs1410996 (intron 15), and rs1065489 (exon 19). The most frequent *CFH* haplotype was the CGTAAG (H4a haplotype), with a frequency of 0.492 in cases and 0.638 in “supercontrols,” followed by the TGTAAG (H4b haplotype, Table 4), with no difference between cases (0.338) and “supercontrols” (0.279). On the contrary, the frequency of the H3 *CFH* haplotype (TGTGGT), which had previously been associated with aHUS (15) was very low and estimated to be 0.024 in cases and 0.01 in “supercontrols” (*P*-value = 0.04, Table 4). Notably, the frequency of the *CFH* H3 haplotype in aHUS patients without anti-FHs of our cohort (*n* = 275) was 0.292 and was significantly higher compared to cases (*P*-value = 0.0002), “supercontrols” (*P*-value = 5.6 × 10^−8^) as well as the general population (H3 haplotype frequency = 0.203, among 2,504 controls from 1,000 genomes project, *P*-value = 1.85 × 10^−6^).

**Table 4:** Estimated *CFH* haplotypes in the 25 cases and the 48 “supercontrols.”

**Estimated *CFH* haplotype**	**Haplotype reference number**	**Haplotype frequency**		***P*-value**
		**Cases**	**Supercontrols**	
CGTAAG	H4a	0.492	0.638	ns
TGTAAG	H4b	0.338	0.279	ns
CATAAG	H2	0.07	0.019	ns
TGCAGG	H5	0.042	0	ns
TGTGGT	H3	0.024	0.01	0.04

*The estimated allele frequencies of each CFH haplotype in cases (aHUS patients with anti-FHs and FHR1 deficiency) and “supercontrols” (adult healthy subjects with homozygous CFHR1 deletion) were shown. The minimal informative SNPs within CFH gene considered for this analysis were rs3753394, rs800292, rs1061170, rs3753396, rs1410996, and rs1065489*.

In summary, no significant association has been found between *CFH* haplotypes and anti-FHs mediated aHUS, using “supercontrols” as the reference group.

In our cohort of aHUS patients with anti-FHs, the H4a and H4b *CFH* haplotypes were in strong LD with the *CFHR3-CFHR1* deletion (Table 5), which is consistent with published data (16), while the H5 and H3 haplotypes were associated with the *CFHR1-CFHR4* deletion (Table 5).

**Table 5:** Linkage disequilibrium between *CFH* haplotypes and *CFHR3-CFHR1* or *CFHR1-CFHR4* deletions in the 30 aHUS patients with anti-FHs.

**Estimated *CFH* haplotype**	**Haplotype reference number**	***CFHR3-CFHR1* deletion**	***CFHR1-CFHR4* deletion**	**Frequency mean**
CGTAAG	H4a	Yes	No	0.504
TGTAAG	H4b	Yes	No	0.329
CATAAG	H2	Yes	No	0.059
TGCAGG	H5	No	Yes	0.042
TGTGGT	H3	No	Yes	0.021

*Yes, deleted allele; No, normal allele. The last right column shows the estimated frequencies of the extended haplotypes including CFH and the CFHR3-CFHR1-CFHR4 region*.

The frequency of the *CD46* c.^*^783C allele (rs7144) that tags the *CD46*_GGAAC_ aHUS risk haplotype (52), did not differ between cases (0.38) and “supercontrols” (0.35, Table 3). On the contrary, the frequency of the C allele was significantly higher in our aHUS patients without anti-FHs (0.45) as compared with the 2,504 controls (allele frequency: 0.35) from the 1,000 genomes project (*P*-value = 0.001).

Moreover, we studied the prevalence of common polymorphisms in *C3* c.941C>T, p.Pro314Leu (rs1047286), *C3* c.304C>G, p.Arg102Gly (rs2230199), *CFB* c.94C>T, p.Arg32Trp (rs12614), *CFB* c.95G>A, p.Arg32Gln (rs641153) and *THBD* c.1418C>T, p.Ala473Val (rs1042579), which were previously reported in association with other immune-mediated diseases, including C3 glomerulopathy (33, 34), IgA nephropathy (35), and age-related macular degeneration (36–38). No significant difference was found with regard to allele frequencies of any of the above single nucleotide polymorphisms (SNPs) between cases and “supercontrols” (Table 3).

Finally, we analyzed the combination of the three variants FH Val62, FB Arg32, and C3 102Gly, which in functional studies (53, 54) have been associated with higher C3 convertase activity (risk complotype), and we did not find any difference in the prevalence of the risk complotype between cases (0.24) and “supercontrols” (0.17, *P*-value = 0.39).

### Genetic Screening of *CFH* Promoter

**Figure 6:**
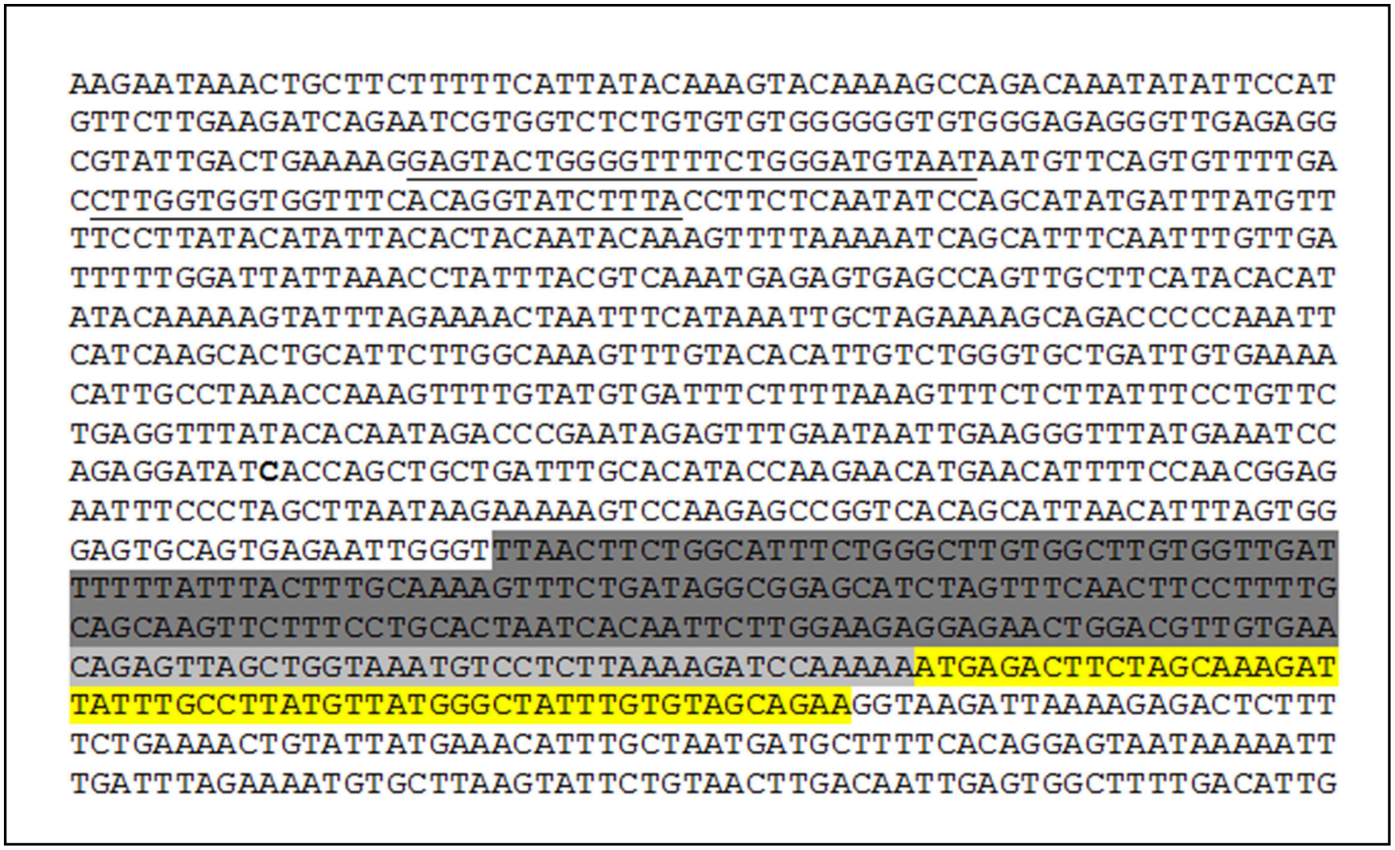
*CFH* promoter sequence. Exon 1 (yellow), basal promoter (light gray), proximal promoter (dark gray), and two AIRE consensus sequences predicted by Matinspector software–Genomatix (underlined) are shown.

We then wondered whether rare variants in the *CFH* promoter that could affect FH expression in the thymus may predispose to a lack of central tolerance toward FH and to the development of anti-FHs HUS. The analysis through Matinspector software (Genomatix) revealed the presence of two sequences predicted with a good score as consensus motifs for the “autoimmune regulator” transcription factor (AIRE, Figure 6), a DNA binding molecule that is involved in central thymic tolerance by promoting the expression of tissue-specific antigens in medullary thymic epithelial cells. We sequenced the c.1-1070 region upstream the *CFH* gene, including the two predicted AIRE consensus regions, in our aHUS patients with anti-FHs.

We did not find any rare variant (with MAF ≤ 0.01) in the two DNA sequences predicted as AIRE consensus regions or in the entire sequenced *CFH* promoter region.

### Clinical and Biochemical Features at Disease Onset

Clinical and biochemical features at onset for all the 30 patients with anti-FHs are reported in Table 6.

**Table 6:** Clinical and biological data at onset of the 30 patients with anti-FH associated aHUS.

**ID**	**Sex**	**Ethnicity**	**Population**	**Disease onset**						
				**Age (years)**	**Disease triggers**	**Hb (14–18 g/dl)**	**LDH (266–500 U/L)**	**Platelets (150–400 × 10^**3**^/ul)**	Creatinine (0.5–0.8 mg/dl)^*^	**Dialysis (yes/no)**
1	F	Caucasian (Eu)	Italian	7.0	Vomiting	4.5	na	38,000	2.7	Yes
2	M	Caucasian (Eu)	Italian	7.7	Flu like	6.3	4,185	64,000	1.6	No
3	F	Caucasian (Eu)	Portuguese	7.6	Resp. infection	8.5	538	78,000	4.9	Yes
4	F	Caucasian (Eu)	German	8.0	None	6	2,464	16,000	1.56	Yes
5	M	Caucasian (Eu)	Italian	8.8	Vomiting	6	na	167,000	9.4	Yes
6	M	Caucasian (Eu)	Italian	6.2	Flu like	6.9	na	30,000	6.9	Yes
7	M	Caucasian (Eu)	Italian	5.7	None	6.5	3,650	58,000	2.4	No
8	M	Caucasian (USA)	American	6.7	na	na	na	na	na	Yes
9	F	Caucasian (Eu)	Serbian	7.6	Vomiting	10.4	5,120	48,000	7	Yes
10	F	Caucasian (Eu)	Serbian	6.6	Resp. infection	6	2,022	124,000	1.25	No
11	F	Caucasian (Eu)	Italian	5.4	Vomiting	7	2,930	42,000	2.8	Yes
12	M	Caucasian (Eu)	Italian	7.8	Vomiting	6.8	2,866	96,000	1.9	Yes
13	F	Caucasian (Eu)	Turkish	12.8	None	10.6	1,767	12,000	2.6	No
14	F	Caucasian (Eu)	Bulgarian	15.5	Vomiting, fever	9	3,597	79,000	3.7	Yes
15	M	Caucasian (Eu)	Polish	11.0	Vomiting	7.8	1,908	43,000	4.3	Yes
16	F	Caucasian (USA)	American	10.5	Vomiting, fever	8	na	29,000	11	Yes
17	F	Caucasian (USA)	American	5.3	Flu like	8	1,422	120,000	3	Yes
18	M	Caucasian (Eu)	Italian	10.1	Vomiting	5.2	3,770	70,000	7	No
19	F	Caucasian (Eu)	Italian	8.8	na	7.2	na	60,000	4	Yes
20	F	Caucasian (Eu)	Dutch	30.2	Cesarean	9.5	3,362	40,000	1.8	No
21	F	Hispanic	Argentine	17.6	Oral contraceptive	na	na	na	na	Yes
22	M	Caucasian (Eu)	Polish	7.6	Resp. infection	9	na	na	9	Yes
23	M	Asian	Japanese	9.2	Vomiting, fever	6.5	2,569	15,000	2	Yes
24	M	Jewish	Israeli	5	None	4.8	1,091	76,000	1.5	Yes
25	M	Persian	Iranian	15.0	Resp. infection	8	7,752	23,000	5.7	Yes
26	M	Hispanic	Argentine	6.5	None	9	na	18,000	3.5	Yes
27	M	Caucasian (Eu)	Italian	7.1	Resp. infection	6.6	na	52,000	4.7	Yes
28	F	Caucasian (Eu)	Italian	8.1	Vomiting, fever	na	na	na	na	Yes
29	M	African-Arab	Yemenian	1.3	Resp. infection	5	1,916	150,000	0.6	No
30	M	Caucasian (Eu)	Belorussian	6.9	Vomiting	5.8	5,619	62,000	2.33	Yes
				**Median [IQR]**		**Mean ± SD**	**Mean ± SD**	**Mean ± SD**	**Mean ± SD**	**% Yes**
		**Overall**		7.6 [6.6–9.9]		7.3 ± 1.4	3,081.5 ± 1,736	61.923 ± 41,218	5.7 ± 3.3	77
		**FHR1 deficiency**	Yes	7.6 [6.6–9.2]		7.1 ± 1.3	3,010 ± 1,696	66,272 ± 42,332	6.5 ± 3.4	76
			No	8.8 [6.9–12.8]		8.2 ± 2.1	3,693.0 ± 2,723.8	38,000 ± 26,683	3.3 ± 1	80
			*P*-value	0.4		0.23	0.61	0.21	0.4	0.7
		**LPVs**	Yes	7.1 [5.7–8.8]		5.6 ± 0.9	2,521.8 ± 1,325.0	62,750 ± 40,679	3.3 ± 1	56
			No	7.8 [6.9–10.1]		7.6 ± 1.3	3,281.4 ± 1,832.7	61,555 ± 42,621	6.5 ± 3.4	86
			*P*-value	0.32		0.34	0.42	0.95	0.38	0.18

The Patient ID, the sex (F, female; M, male), the ethnicity, the population of origin, the age at the disease onset, the observed triggers, hemoglobin (Hb), lactate dehydrogenase (LDH), platelet count, serum creatinine and the need for dialysis are shown. na, not available. Normal values are in brackets.

* *Normal values for children between 5 and 10 years. For children < 1–5 years, 0.3–0.5 mg/dl; for children >10 years/adults, 0.5–1.2 mg/dl*.

**Figure 7:**
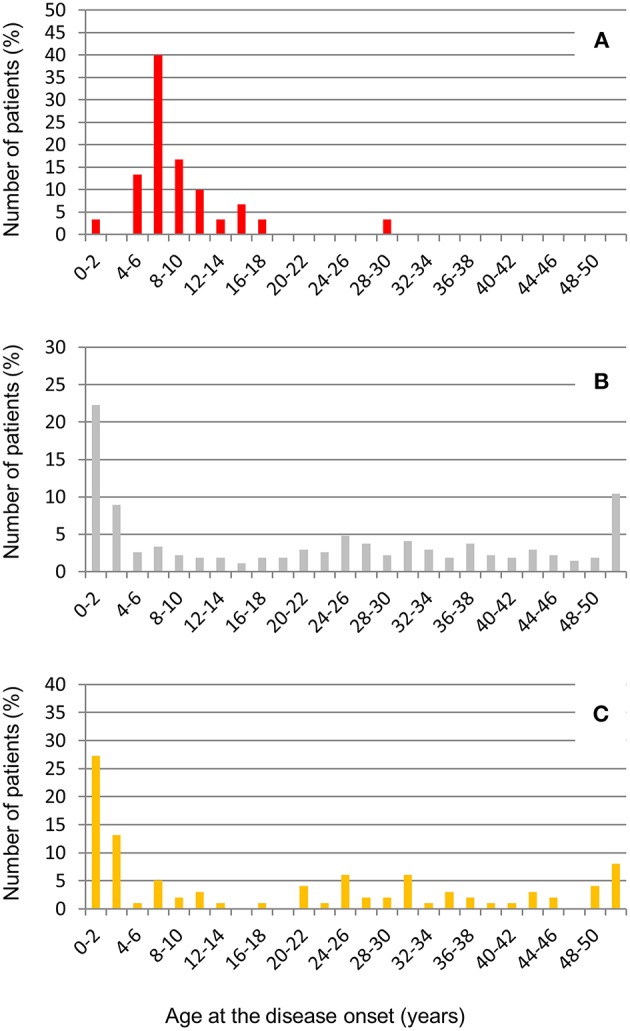
Distribution of age at disease onset in aHUS patients analyzed for anti-FHs. **(A)** Patients with anti-FHs (*n* = 30); **(B)** Patients without anti-FHs (*n* = 275), and **(C)** Patients without anti-FHs and carrying LPVs in complement genes (*n* = 99).

The large majority of patients with anti-FHs in our cohort were Caucasian (53% males, Table 6). All but one developed aHUS during childhood or adolescence (96.7%). The median age of disease onset was 7.6 [6.6–9.9] years (Table 6). The outliers were a woman who developed anti-FHs aHUS at 30 years of age after a cesarean delivery and a child who manifested aHUS at 16 months (he also carried a *THBD* LPV). The distribution of the age at disease onset in patients with anti-FHs was unimodal, with a sharp incidence peak around 8 years of age (Figure 7A), which was significantly different from aHUS patients negative for anti-FHs who exhibited a bimodal distribution, with an early peak before 4 years of age, and a late peak during adulthood (Figure 7B). Similar bimodal distribution was observed when we considered only patients negative for anti-FHs and carrying complement gene abnormalities (in *CFH, MCP, CFI, THBD, C3*, and *CFB;* Figure 7C).

Through logistic regression we estimated that the homozygous deletion of *CFHR1* and age at disease onset between 4 and 12 years were strongly associated with the risk for an aHUS patient to present anti-FHs. Twenty-four out of 30 aHUS patients with anti-FHs (80%) vs. 25 out of 275 aHUS patients without anti-FHs (9%) had the homozygous deletion of *CFHR1* (OR [95% CI] = 40 [14.9–107.1], *P*-value = 1.4 × 10^−22^, Table 7). Twenty-four out of 30 aHUS patients with anti-FHs (80%) vs. 27 out of 275 aHUS patients without anti-FHs (9.8%) developed the disease between 4 and 12 years of age (OR [95% CI] = 37 [13.8–97.9], *P*-value = 1.7 × 10^−21^, Table 7). The combination of the homozygous *CFHR1* deletion and age at disease onset between 4 and 12 years further increased the risk to have anti-FHs aHUS (OR [95% CI] = 108 [33.7–346.5], *P*-value = 6.9 × 10^−33^, Table 7).

**Table 7:** Estimate of the risk of an aHUS patient having anti-FHs in the presence of *CFHR1* homozygous deletion and/or age at disease onset between 4 and 12 years.

		**Anti-FHs**		**Sensitivity**	**Specificity**	**OR [CI 95%]**	***P*-value**
		**Positive**	**Negative**				
Hom *CFHR1*Δ	Yes	24	25	0.80	0.91	40 [14.9–107.1]	1.4 × 10^−22^
	No	6	250				
Onset 4–12 years		24	27	0.80	0.90	37 [13.8–97.9]	1.7 × 10^−21^
Onset < 4 or ≥13 years		6	248				
Hom *CFHR1*Δ and onset at 4–12 years	Yes	20	5	0.67	0.98	108 [33.7–346.5]	6.9 × 10^−33^
	No	10	270				

*OR, odds ratio*.

Prodromal signs were observed in most (70%) anti-FH positive patients: twelve patients (40%) exhibited gastrointestinal symptoms (four of them with fever), and nine patients (30%) had upper respiratory tract infections (including flu like symptoms). One patient developed aHUS post-partum and another while on oral contraception. Five patients did not have any prodromal signs or triggers and for two patients trigger data were not available (Table 6). At disease onset, all patients showed anemia, thrombocytopenia, high LDH levels, and high serum creatinine. Twenty-three (77%) patients developed kidney failure requiring dialysis (Table 6).

Among patients with anti-FHs, we did not find any significant difference in the age of onset and in clinical complement parameters between cases with FHR1 deficiency and patients with one or two copies of *CFHR1* (Table 6), as well as between patients with or without LPVs (Table 6).

### Complement Profile

Data on serum C3 and C4 levels at the time of anti-FH antibody assay were available for 28 patients (Table 8). Fourteen patients (46.7%) had lower than normal serum C3 levels, in most of them C3 was measured during convalescence (Table 8). C4 levels, on the contrary, were normal in all patients (Table 8). Plasma FH plus FHL1 levels were in the normal range in all patients (Table 8) with the only exception being patient 14 (studied in remission and with a high antibody titer, 1,557.3 AU/ml) who had 80 mg/l FH (Table 8). Plasma FH plus FHL1 levels did not differ between patients studied during the acute episode and patients studied in remission (acute: 340.3 ± 49.3 mg/l *n* = 3; vs. remission: 344.7 ± 117.1 mg/l, *n* = 21; ANOVA, *P*-value = 0.951) and did not correlate with the antibody titer (r^2^ = 0.0085, *P*-value = ns). The latter finding would indicate that the antibodies used in our FH ELISA assay were detecting total FH, including free FH and immune-complexed FH (55), which is consistent with previously published studies (17, 56).

**Table 8:** Serum C3, C4, and plasma FH + FHL1 levels evaluated at the time of anti-FH measurement.

**Patient ID**	**Biochemical complement parameters**				
	**Disease phase**	**Anti-FH titer (AU/mL)**	**C3 level (90–180 mg/mL)**	**C4 level (10–40 mg/mL)**	**FH+FHL1 level (172–507 mg/L)**
1	Rem	453.4	30	12	na
2	Rem	381.6	141	27	na
3	Rem	280.8	112	29	na
4	Rem	513.2	na	na	na
5	Rem	146.6	85	25	349
6	Rem	243.0	81	26	309
7	Rem	572.0	94	17	312
8	Rem	1,646.1	77	31	283
9	Rem	876.7	70	19	302
10	Acute	1,314.9	103	25	397
11	Acute	2,781.0	96	28	na
12	Rem	342.9	185	62	307
13	Rem	1,590.4	118	22	686
14	Rem	1,557.3	35	27	80
15	Rem	862.3	100	30	438
16	Rem	877.4	na	na	308
17	Rem	189.15	106	70	446
18	Rem	531.2	75	14	463
19	Rem	226.6	78	20	374
20	Rem	111.8	149	38	432
21	Rem	96.6	39	25	244
22	Rem	102.1	89	39	362
23	Acute	123.1	54	29	307
24	Rem	77.6	126	35	226
25	Acute	440.15	95	27	317
26	Rem	141.2	96	27	362
27	Rem	65.2	65	14	246
28	Rem	57.1	99	22	372
29	Rem	303.9	68	11	na
30	Rem	1,321.7	63	31	337
		**Mean ± SD**	**Mean ± SD**	**Mean ± SD**	**Mean ± SD**
	Acute (*n* = 4)	1,164.8 ± 1,189.5	87 ± 22.3	27.2 ± 1.7	340.3 ± 49.3
	Rem (*n* = 26)	521.8 ± 504.3	90.9 ± 36.3	28 ± 14	344.7 ± 117.1

*The phase of the disease when the samples were collected (Rem, remission phase; Acute, acute phase), the anti-FH titer, and serum C3, C4 levels and plasma FH **+** FHL1 levels are shown. Normal ranges are in brackets; na, not available*.

### Clinical Outcome

During a median of 36 months follow-up (IQR = 12–72 months), 11 patients with anti-FHs experienced relapses of the disease (37%): 9 had the FHR1 deficiency, one the FHR1 deficiency plus a LPV in *CFH* and one a LPV in *CD46* and 1 copy of *CFHR1*. Overall, 15 patients (50%) developed end stage renal disease (ESRD): 8 had the FHR1 deficiency, 2 the FHR1 deficiency plus a LPV in *C3* and *CFH*, respectively and 2 a LPV in *THBD* (and 1 copy of *CFHR1*) and *C3* (and 2 copies of *CFHR1*), respectively. The prevalence of relapses or ESRD during the follow-up did not differ statistically between patients with or without LPVs (relapses: patients with LPVs 22%, without LPVs 43%; *P*-value = 0.42, ESRD: patients with LPVs 44%, without LPVs 52%; *P*-value = 1.0).

Four patients received kidney transplantation and aHUS recurrence was observed in one of them, who also carried the C3 heterozygous LPV p.Arg592Trp. Another patient died following the transplant due to clinical complications unrelated to aHUS.

## Discussion

Here we report the results of a retrospective study in a large cohort of patients with aHUS in which we described the clinical and genetic features of 30 patients with anti-FHs.

Through combined CNV analysis and *CFHR1* sequencing we confirm that the FHR1 deficiency is strongly associated with anti-FHs aHUS. Moreover, through an approach based on the inclusion of “supercontrols,” we demonstrate that patients with anti-FHs are enriched in complement gene LPVs, while common complement gene variants known to increase the risk of aHUS or other immune -mediated diseases do not significantly contribute to anti-FHs aHUS.

The prevalence of anti-FHs in our cohort of aHUS patients (10%) is consistent with data from other European cohorts (5–13%) (28), but is lower than that observed in an Indian aHUS cohort in which the percentage of anti-FH positive patients was dramatically higher up to 56.1% (57).

Finding that 83% of our aHUS patients with anti-FHs had a complete FHR1 deficiency due to the homozygous *CFHR3-CFHR1* deletion or, more rarely, to compound heterozygous *CFHR3-CFHR1* and *CFHR1-CFHR4* deletions is consistent with earlier published studies in other cohorts (21, 22). Interestingly, we describe a FHR1-deficient patient carrying the *CFHR1* deletion in one allele and a frameshift *CFHR1* mutation causing an early termination of protein translation in the other allele. To the best of our knowledge only one other aHUS patient with anti-FHs and a truncating *CFHR1* mutation has been described in a published study (21). However, we found 5 patients with anti-FHs carrying at least one normal copy of *CFHR1*, indicating that autoantibodies against FH can also be formed in the presence of FHR1.

In agreement with previous reports (24–27, 58), anti-FHs from 92% of our patients predominantly targeted the C terminus of FH, which is a mutational hot spot in aHUS and a crucial domain involved in the regulation of the complement AP on cell surface (59). Our results, showing that serum from two patients with anti-FHs and no LPVs in complement genes induced higher than normal C5b-9 deposits on cultured endothelial cells, support a pathogenetic role of anti-FHs in causing complement dysregulation on endothelium (58, 60). This finding is in line with previous studies showing that aHUS-associated anti-FH autoantibodies impaired FH capability to protect rabbit or sheep erythrocytes from complement-mediated lysis (25, 61).

The mechanisms that link the lack of FHR1 to the development of anti-FHs have not been clarified yet. Evidence that the FH C-terminal domain is homologous to FHR1 C-terminus (62) and that most anti-FHs cross-react with SCRs 4–5 of FHR1 (22, 26) suggested that the absence of FHR1 plays a role in the loss of tolerance to FH (28). In the *CFH* promoter region we identified two sequences predicted as possible consensus site for the binding of AIRE, a transcription factor that is crucial for the expression of tissue-specific antigens in the thymus and the maintenance of central tolerance (63–65). However, the failure to find any variant in the *CFH* promoter in our patients with anti-FHs aHUS does not support the hypothesis that there is a defect of central tolerance related to AIRE in anti-FHs development.

Of relevance to understand the immunologic basis of anti-FHs, are data that the large majority of patients with anti-FHs of our cohort had prodromal infectious illnesses, most commonly upper respiratory tract and gastrointestinal infections, and developed the disease between 4 and 12 years of age, which corresponds to the peak of incidence of common infections. These findings, together with published reports showing a high prevalence of respiratory tract infections (19, 66), and gastrointestinal pathogens in aHUS patients with anti-FHs (67), would support a “two-hit” model according to which the autoimmunity toward FH could develop as a result of an infection in subjects with genetic predisposing background (28). Bhattacharjee et al. (26) identified an anti-FH autoantibody epitope cluster within SCR20, which includes amino acids used by microbes to bind FH and evade the immune system (68–70). The authors proposed that binding of this domain with certain microbes induces a conformation change in FH SCR20 generating a neoepitope similar to FHR1, which might predispose to the development of the autoantibodies against FH in subjects with the FHR1 deficiency.

Anti-FHs aHUS is an ultra-rare disease with a prevalence of about 1/1,000,000 people in Europe and the USA (1, 28) which does not fit with the ~5% prevalence of FHR1 deficiency in European Caucasian populations (29), and with bacterial infections that commonly occur during school-age (67). From these observations we infer that multiple genetic and/or environmental risk factors are required for the disease to manifest, as reported for other autoimmune disorders (71).

Recently, as a new strategy to identify genetic variants associated with complex traits, patients were compared with centenarians, as supercontrols, who have evaded all the common diseases associated with aging and are expected to be depleted in disease predisposing genetic variants or enriched in protective variants (72, 73). Inspired by these reports, to identify other anti-FHs aHUS genetic determinants, here we adopted a similar approach using as “supercontrols” adults subjects with the homozygous *CFHR1* deletion who did not get anti-FHs aHUS despite carrying the known genetic risk factor for the disease (i.e., FHR1 deficiency) and likely having been exposed to common infections during childhood. We hypothesized that the comparison of our patients with anti-FHs aHUS and FHR1 deficiency (cases) with “supercontrols” could allow us detecting genetic variants with clinical value at relatively small sample size. With this approach we demonstrate that cases are significantly enriched in *CFH, THBD* and *C3* LPVs compared to “supercontrols.” Other authors have screened patients with anti-FHs aHUS for complement gene defects, but there was no comparison with a control group and the results were conflicting (18, 19, 21–23, 32). Our data support the hypothesis that rare complement gene variants are involved in predisposing to anti-FHs aHUS. *CFH* LPVs are the most abundant in our cases, two of which are not reported in public control databases and are predicted to be strongly damaging *in silico*. The third *CFH* LPV, the p.Gln950His, was also found in 4 patients without anti-FHs of our aHUS cohort and in other published patients (22, 44–46). The functional relevance of this variant was documented by Mohlin and colleagues (44) who showed that the 950His variant is less effective in inhibiting complement-mediated sheep erythrocyte lysis than the wild type Gln950. As for the *THBD* variants, both have been found to be less effective in promoting C3b inactivation to iC3b on the cell surface (12). Finally, the *C3* p.Gly637Arg has not been described in patients with aHUS, it is predicted to be strongly damaging by *in silico* analysis but functional data are not available. We would speculate that complement LPVs contribute to aHUS in subjects with FHR1 deficiency by synergizing with anti-FHs in inducing AP activation on cell surfaces. Since complement activation products can enhance the adaptive immune response (74), the possibility that AP dysregulation associated with LPVs *per se* could favor the proliferation of the self-reactive T cell clones and the formation of anti-FHs is worth investigating. However, further studies are required to clarify the mechanisms through which LPVs predispose to anti-FHs aHUS.

We found only one LPV in the “supercontrol” group, namely the p.Arg317Trp in FI. This variant was previously reported to have a lower cofactor activity compared to wild type FI (50). However, later on Nilsson and colleagues found that the activity of the 317Trp variant was not impaired in any functional assay, rather this variant was more efficient than wild type FI in C3b cleavage on the surface of endothelial cells (51).

At variance with LPVs, common genetic variants in complement genes predisposing to aHUS or to other immune-mediated diseases (as C3 glomerulopathy, age-related macular degeneration, and IgA nephropathy) (33–38) were not more abundant in our cases compared to “supercontrols.” Specifically, the *CFH* H3 haplotype, which has been strongly associated with an increased risk of aHUS (15), was rare both in cases and in “supercontrols,” although it was slightly more prevalent in cases. As expected, this haplotype was enriched in our patients without anti-FHs. These results do not surprise, because most cases and “supercontrols” share the *CFH* H4 haplotypes that are in LD with the *CFHR3-CFHR1* deletion. As for the frequency of the *CD46* c.^*^783C allele (rs7144) variant that tags the *CD46*_*GGAAC*_ haplotype, described in strong association with aHUS (52), we did not observe any difference between cases and “supercontrols,” whereas the frequency of this allele was significantly enriched in our aHUS patients without anti-FHs. Altogether our data demonstrate that the *CFH* H3 and the *CD46*_*GGAAC*_ haplotypes are risk factors for aHUS without anti-FHs but do not contribute to development of anti-FHs aHUS.

The marked difference we observed in the age of onset between patients with anti-FHs and those negative for anti-FHs further supports the hypothesis that diverse predisposing factors underlying the two forms of the disease are involved.

Importantly, within the entire aHUS cohort reported here we have shown that a disease onset between ages 4 and 12, identifies the presence of anti-FHs with a specificity of 90%. Patients with the above age of onset should be screened carefully for anti-FHs. Prompt identification of anti-FHs is of great clinical relevance since anti-FHs can be removed by successfully plasma exchange, which also supplements free FH and provides FHR1, which might act as a decoy for the antibodies (75). Patients should be followed up carefully long-term to monitor the re-emergence of anti-FHs, since this form of aHUS is associated with a high prevalence of relapses, as documented by published (28) and present data. Relapses can be prevented by maintenance therapy with immunosuppressive agents that inhibit the further production of anti-FH antibodies (28), but these drugs may have serious side effects particularly in children.

In conclusion, we have confirmed in our cohort of patients, the strong association between FHR1 deficiency and aHUS with anti-FH autoantibodies. Through an innovative approach based on the comparison with “supercontrols” carrying the homozygous *CFHR1* deletion, identified by screening a large number of healthy adult subjects, we have documented that patients with anti-FHs aHUS are enriched in complement gene LPVs. This observation indicates that the pathogenesis of anti-FHs aHUS is complex and multiple “hits” are required for its clinical manifestation. We also document that the *CFH* H3 and the *CD46*_*GGAAC*_ haplotypes are not associated with anti-FHs aHUS, whereas these haplotypes are enriched in aHUS patients without anti-FHs, which highlights the differences in the genetic basis of the two forms of the disease. Finally, we confirm the role of common infections as environmental factors that contribute to the development of anti-FHs aHUS in genetically predisposed individuals. The latter finding fits with the sharp peak of disease onset during scholar-age. Further studies are needed to fully elucidate the genetic and environmental factors underlying anti-FHs aHUS and to establish whether the combination of anti-FHs with LPVs or other risk factors influences the course of the disease and the response to therapies.

## Ethics Statement

All subjects provided informed written consent in accordance with the Declaration of Helsinki. The study was approved by the Ethics Committee of Azienda Sanitaria Locale, Bergamo, Italy.

## Author Contributions

EV, MN, and GR designed research, interpreted data, and wrote the paper. EV, MA, PI, RP, CM, MB, AC, RD, and SA performed the research and analyzed the data. EB provided detailed clinical information of patients. AB and AA analyzed the data and critically revised the manuscript.

### Conflict of Interest Statement

MN has received honoraria from Alexion Pharmaceuticals for giving lectures, and for participating in advisory boards and research grants from Omeros, Alnylam, and Chemocentryx. GR has consultancy agreements with AbbVie^*^, Alexion Pharmaceuticals^*^, Bayer Healthcare^*^, Reata Pharmaceuticals^*^, Novartis Pharma^*^, AstraZeneca^*^, Otsuka Pharmaceutical Europe^*^, Concert Pharmaceuticals^*^. ^*^No personal remuneration is accepted, compensation is paid to his institution for research and educational activities. None of these activities have had any influence on the results or interpretations in this article. The remaining authors declare that the research was conducted in the absence of any commercial or financial relationships that could be construed as a potential conflict of interest.
